# Cascade polymerizations: recent developments in the formation of polymer repeat units by cascade reactions

**DOI:** 10.1039/d0sc01475c

**Published:** 2020-04-29

**Authors:** Gregory I. Peterson, Tae-Lim Choi

**Affiliations:** Department of Chemistry, Seoul National University Seoul 08826 Republic of Korea gpeterson@snu.ac.kr tlc@snu.ac.kr

## Abstract

Traditionally, most polymerizations rely on simple reactions such as alkene addition, ring-opening, and condensation because they are robust, highly efficient, and selective. These reactions, however, generally only yield a single new C–C or C–O bond during each propagation step. In recent years, novel macromolecules have been prepared with propagation steps that involve cascade reactions, enabling various combinations of bond making and breaking steps to form more complex repeat units. These polymerizations are often challenging, given the requirements for high conversion and selectivity in controlled polymerizations, yet they provide polymers with unique chemical structures and significantly broaden the scope of how polymers can be made. In this perspective, we summarize the recent developments in cascade polymerizations, primarily focusing on single-component cascades (rather than multi-component polymerizations). Polymerization performance, monomer scope, and mechanisms are discussed for polymerizations utilizing radical, ionic, and metathesis-based mechanisms.

## Abstract 2

Cascade polymerizations provide new routes to unique polymer structures. In this perspective, we summarize their performance, monomer scope, and mechanisms.

## Graphical abstract

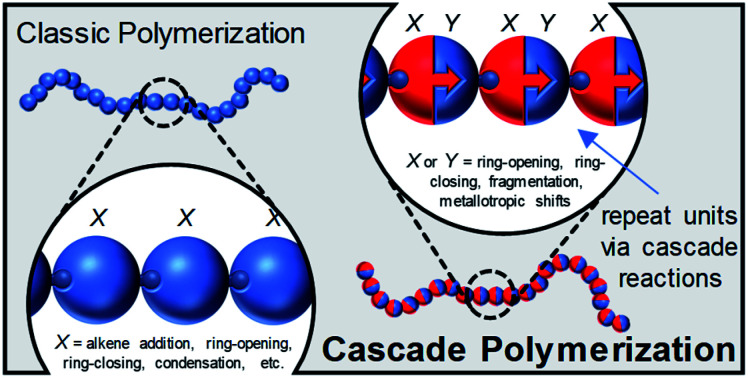

## Introduction

Organic chemists have devoted considerable effort to the development of reactions in which a series of chemical transformations occur (with multiple bonds being formed and/or broken) without the requirement of isolating intermediates or significantly changing reaction conditions.^1–6^ Despite efforts to define and classify these types of reactions, they are often indiscriminately termed cascade, tandem, or domino reactions.^1,7–9^ Cascade reactions are often pursued for their increased synthetic efficiency, as they help enable the synthesis of complex molecules with decreased number of reaction and workup steps, as well as potentially reducing the quantities of chemical reagents, solvents, byproducts, and waste. The concept of cascade reactions has also been applied to polymer synthesis (with multiple chemical transformations occurring during the propagation stage) and are often likewise termed cascade, tandem, or domino polymerizations. These polymerizations have been used to prepare a wide range of polymers with novel structures, unique properties, and high potential in various applications.

For this perspective, our aim was to discuss recent developments pertaining to cascade polymerizations. Unfortunately, “cascade” in many cases has been used interchangeably with “tandem” and both terms have been used to describe a wide range of polymerizations with varying propagation mechanisms. In order to clear up this ambiguity, we found it necessary to establish a clear definition of the term “cascade polymerization.” Here we define a cascade polymerization as a polymerization in which the repeat unit is formed *via* a cascade reaction. For the term “cascade reaction” we prefer the definition proposed by Tietze and Beifuss, which has three parts.^9^ First, it is a process involving two or more consecutive reactions (bond-forming or breaking steps) in which subsequent reactions result as a consequence of the functionality formed by the previous step. Second, the overall transformation must take place under the same reaction conditions, without adding additional reagents or catalysts (*i.e.*, it is a “one-shot” polymerization). Third, the preliminary formation of a reactive intermediate (*e.g.*, a carbocation, radical, or metal alkylidene) is not counted as a cascade reaction step. In a chain-growth polymerization, for example, this last part necessitates that after the initiator or polymer chain end reacts with a monomer, at least two more transformations should occur (*e.g.*, ring-opening, ring-closing, fragmentation, *etc.*) to form the final repeat unit.

From here on we use the term “cascade polymerization” regardless of the terminology used in the parent papers. We believe this term has been most consistently applied in the literature to describe polymerizations which are in agreement with our provided definition (the “domino” term has been rarely and inconsistently applied to polymerizations and “tandem” has been used less discriminately than “cascade”). With our strict definition, some polymerizations, which have been classified as cascade polymerizations by others,^10–14^ do not meet the requirements to be classified as such here. Cascade polymerizations can be categorized based on the number of monomers participating in the cascade reaction. Multi-component (*i.e.*, three or more monomers) cascade polymerizations (commonly referred to as just multi-component polymerizations) generally rely on the use of simple mono- and difunctional monomers (for step-growth polymerization), and the desired cascade reaction is achieved by the correct matching of monomer reactivity. In contrast, single-component (*i.e.*, one monomer) cascade polymerizations require the monomer to have multiple reactive functional groups, and thus, success of these polymerizations (*e.g.*, high conversion and selectivity) is largely driven by monomer design.

As multi-component cascade polymerizations have been extensively reviewed in the literature,^15–21^ this review will focus on single-component cascade polymerizations. Despite these polymerizations generally being quite challenging, recently, multiple well-controlled and living cascade polymerizations have been developed, enabling the preparation of new well-defined polymers. Herein, we offer an overview and assessment of cascade polymerizations, primarily focusing on examples from the last two years. We have organized content based on the polymerization mechanisms (radical, ionic, or metathesis-based) and we discuss their polymerization performance, monomer scope, and mechanisms.

### Radical cascade polymerizations

**Fig. 1:**
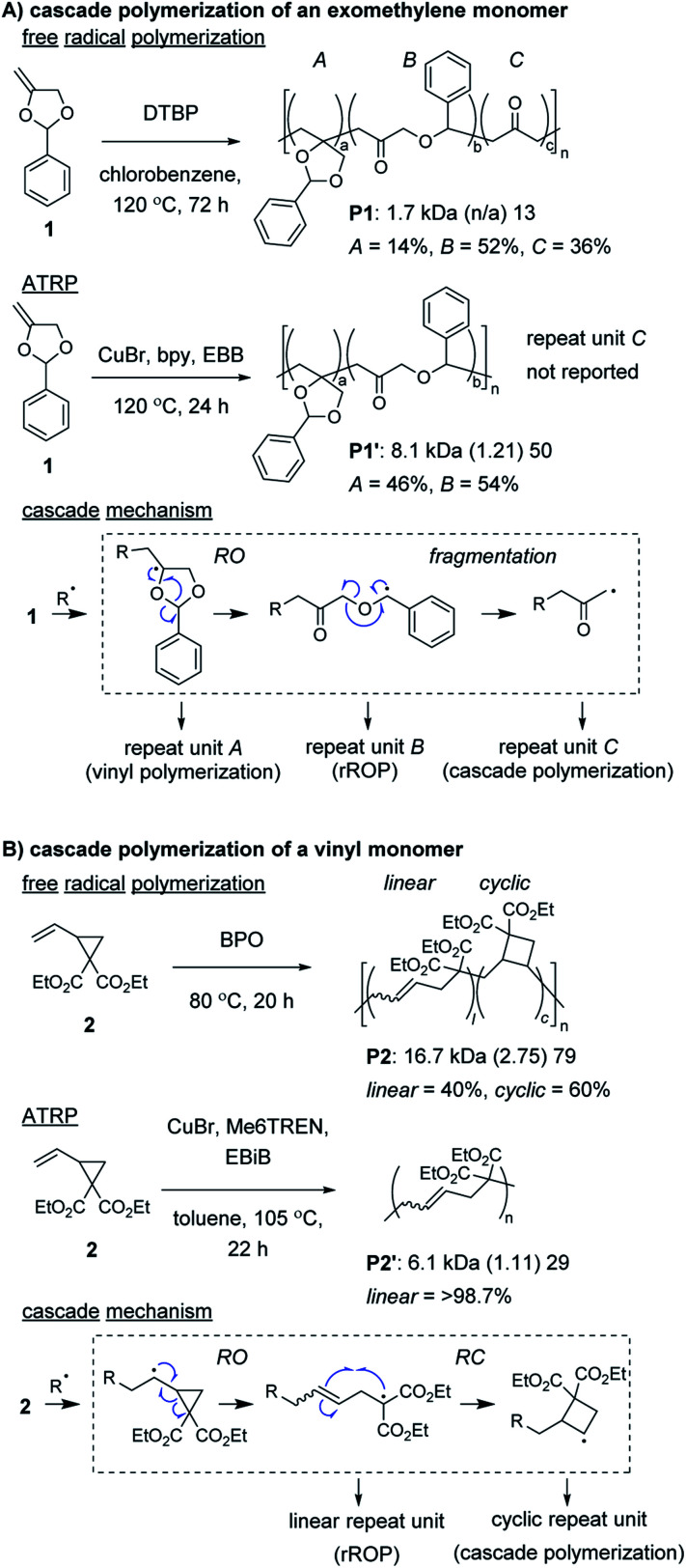
Examples of cyclic (A) exomethylene and (B) vinyl monomers undergoing nonselective cascade polymerization with free radical polymerization conditions or primarily rROP with ATRP conditions. Labels under the polymers = polymer label: *M*_n_ (*Đ*) degree of polymerization (DP).† Abbreviations: DTBP = di-*t*-butyl peroxide. bpy = 2,2′-bipyridine. EBB = ethyl 2-bromobutyrate. BPO = benzoyl peroxide. Me_6_TREN = tris[2-(dimethylamino)ethyl]amine. EBiB = ethyl 2-bromoisobutyrate.

Radical ring-opening polymerization (rROP) is a powerful polymerization methodology used to introduce various functional groups into polymer backbones.^22,23^ Conventional monomers undergo propagation and ring-opening (RO) reactions to generate the repeat units of the polymer. However, depending on the nature of the resulting radical species, further reaction can occur, such as (another) RO, ring-closing (RC), or fragmentation step (thus, meeting the definition of a cascade polymerization). Several rROP monomers based on cyclic exomethylene (*e.g.*, **1**, Fig. 1A) and vinyl (*e.g.*, **2**, Fig. 1B) monomers have been demonstrated to undergo cascade polymerization. Unfortunately, most monomers from these classes have poor polymerization performance (*e.g.*, low conversion, broad molecular weight dispersity, *Đ*) and have poor selectivity for the cascade reaction. For example, under free radical polymerization conditions, **P1** and **P2** were produced with only 36% and 60% of the repeat units (*i.e.*, *C* in Fig. 1A or *cyclic* in Fig. 1B) from the cascade sequence, respectively.^24,25^ Controlled radical polymerization conditions, such as those from atom transfer radical polymerization (ATRP), often further decrease the selectivity for cascade reactions, giving primarily repeat units from rROP (*e.g.*, see **P1′** and **P2′**).^26–28^ Additionally, despite using controlled polymerizations, the resulting polymers generally are limited to number average molecular weight (*M*_n_) values less than 10 kDa. Fortunately, recently developed controlled polymerization conditions and careful monomer design have enabled these issues to be overcome.

**Fig. 2:**
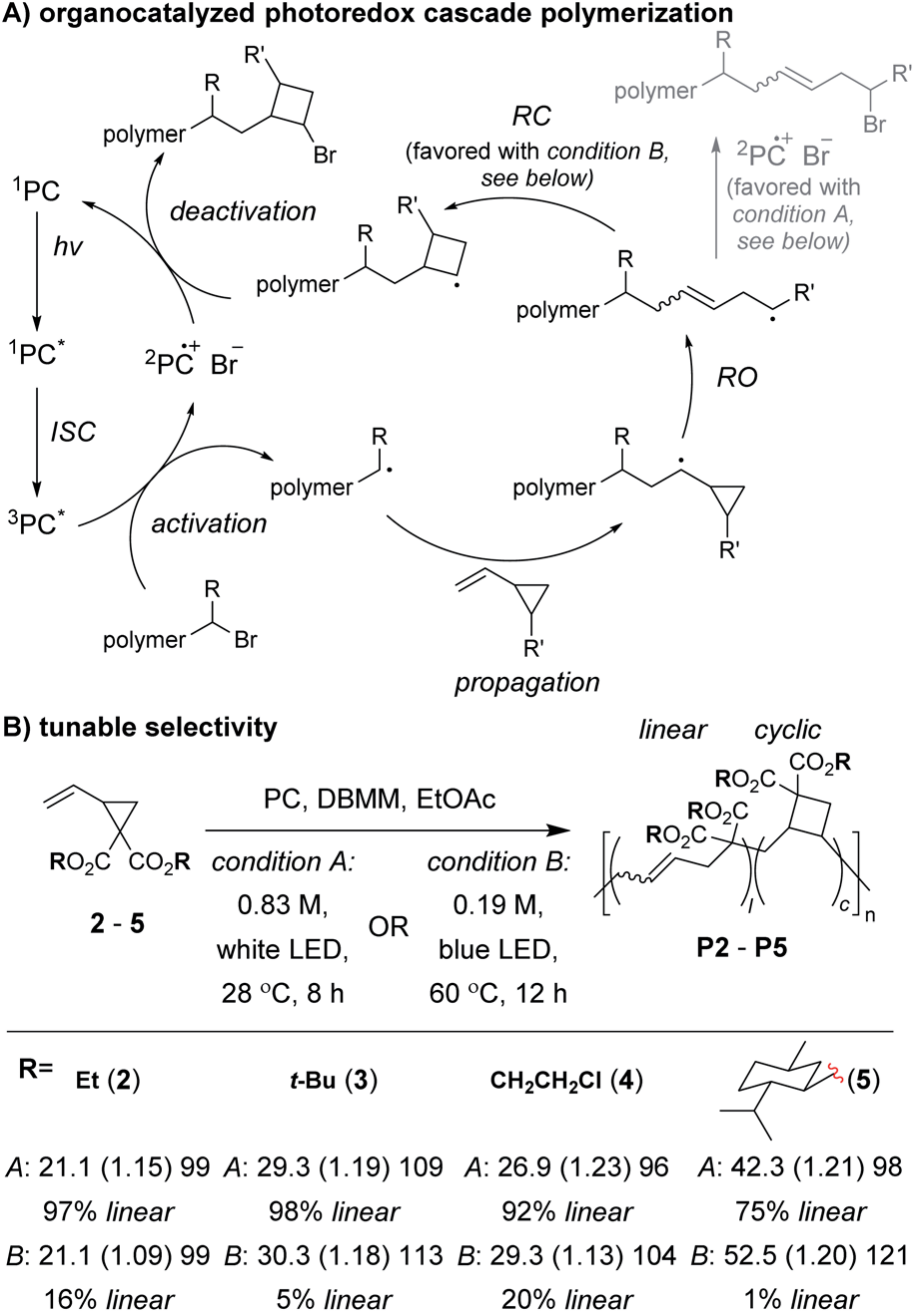
(A) Mechanism of the organocatalyzed photoredox cascade polymerization of vinyl cyclopropanes. (B) The linear *vs.* cyclic repeat unit content could be tuned by changing monomer concentration, LED source, and temperature. Representative examples shown. Labels in part B = *conditions*: *M*_n_ in kDa (*Đ*) DP. Abbreviations: PC = photocatalyst (*N*,*N*-di(2-naphthyl)dihydrophenazine). ISC = inter system crossing. DBMM = diethyl 2-bromo-2-methylmalonate.

Control of the radical species during propagation is essential for successful radical cascade polymerizations. As described above, ATRP of the vinyl cyclopropane monomer **2**, gives low molecular weight polymers which have low cyclic repeat unit content (*i.e.*, poor selectivity for the cascade reaction). Miyake and coworkers proposed†We do not distinguish between *M*_n_ and *Đ* values determined from calibration curves or light scattering methods. DP values were estimated by dividing the reported polymer *M*_n_ (minus end groups if known) by the repeat unit molecular weight.that coordination of the monomer (or its polymer) to the Cu catalyst might inhibit the reversible deactivation of the chain-end radical and would likely be the cause of the poor polymerization performance.^29^ Furthermore, they envisioned that by using organocatalyzed photoredox chemistry (Fig. 2A), they could avoid catalyst coordination, and enable highly controlled polymerizations of vinyl cyclopropanes. They began by first optimizing the polymerization conditions for rROP (specifically, using a *N*,*N*-diaryldihydrophenazine photocatalyst, under white LED irradiation, at a temperature of 28 °C) and achieved controlled living polymerizations with very high selectivity for the linear repeat unit. Remarkably, by decreasing the monomer concentration, using a blue (high power) LED, and increasing the temperature (up to 60 °C), they could almost completely switch the selectivity of the polymerization toward the cascade process and formation of cyclic repeat units (Fig. 2B).

After optimizing conditions for the cascade polymerization, they could tune the monomer-to-initiator ratio (M/I) and obtain **P2** with *M*_n_ values between 21.1 and 105.4 kDa (*Đ* values between 1.03 and 1.16) with 84–87% cyclic repeat units. Furthermore, the controlled and living nature of the polymerization was demonstrated by showing that monomer conversion was strictly controlled by turning on and off the light source (*i.e.*, temporal control) and that a **P2** macroinitiator could undergo chain-extension when re-subjected to the polymerization conditions. Importantly, several other vinyl cyclopropane monomers (*e.g.*, **3–5**) could also undergo cascade polymerization. With bulkier substituents (*e.g.*, **5**), up to 99% selectivity for the cascade reaction was achieved. A limitation to this class of monomer was highlighted by control studies which showed that the polymerization was likely generating mixtures of 4-, 5-, and 6-membered cyclic repeat units. Future work might include further refining the monomer structure to enhance the selectivity for forming a specific ring size and also applying the photoredox chemistry to other rROP monomers which can undergo cascade reactions.

**Fig. 3:**
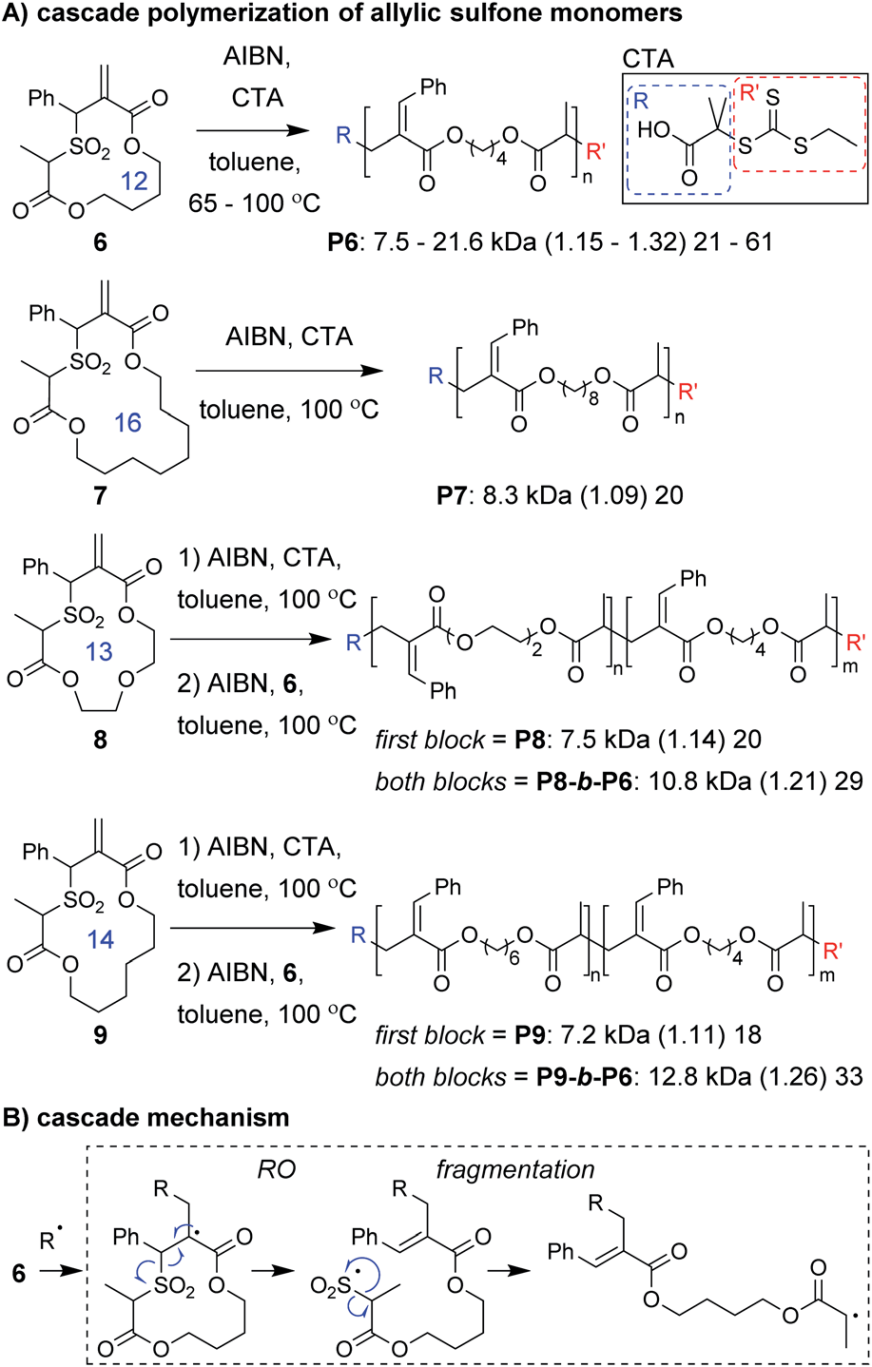
(A) The cascade polymerization of macrocyclic monomers (ring size shown in blue). (B) Mechanism of the RO/fragmentation cascade. Labels under the polymer structures = polymer label: *M*_n_ (*Đ*) DP. Abbreviations: AIBN = azobisisobutyronitrile. CTA = chain transfer agent (structure shown in solid box).

Monomer design is also one of the most important factors for a successful cascade polymerization. Monomers should have a strong driving force for the cascade reaction and should also be compatible with controlled polymerization conditions. Recently, Niu and coworkers developed macrocyclic allylic sulfone monomers (**6–9**, Fig. 3A) that undergo a RO/fragmentation cascade.^30^ These monomers can be contrasted with the structurally related macrocyclic allylic sulfide monomers which generate thiyl radicals that do not undergo reversible deactivation, and are primarily limited to use in copolymerizations with conventional acyclic vinyl monomers.^31–33^ With the new monomers, after RO, SO_2_ extrusion serves as a driving force for the cascade reaction and generates a carbon radical which can undergo reversible deactivation (Fig. 3B). Using reversible addition–fragmentation chain-transfer (RAFT) polymerization conditions, they were able to prepare homopolymers from a 12-membered macrocyclic monomer (**6**) with high molecular weight (up to 21.6 kDa) and narrow dispersity (less than 1.32). Even a 16-membered macrocyclic monomer (**7**) underwent controlled polymerization. Analysis of low molecular weight **P6** by ^1^H NMR spectroscopy and matrix-assisted laser desorption/ionization mass spectrometry (MALDI-MS) confirmed quantitative preservation of the RAFT chain-ends, without evidence of non-selective propagation (*i.e.*, repeat units from propagation prior to fragmentation). The high chain-end fidelity was leveraged to prepare block copolymers derived from macrocyclic monomers of different ring sizes. Specifically, block copolymers with a first block from 13- or 14-membered ring monomers and a second block from a 12-membered macrocyclic monomer were prepared (**P8-b-P6** and **P9-b-P6**, respectively).

**Fig. 4:**
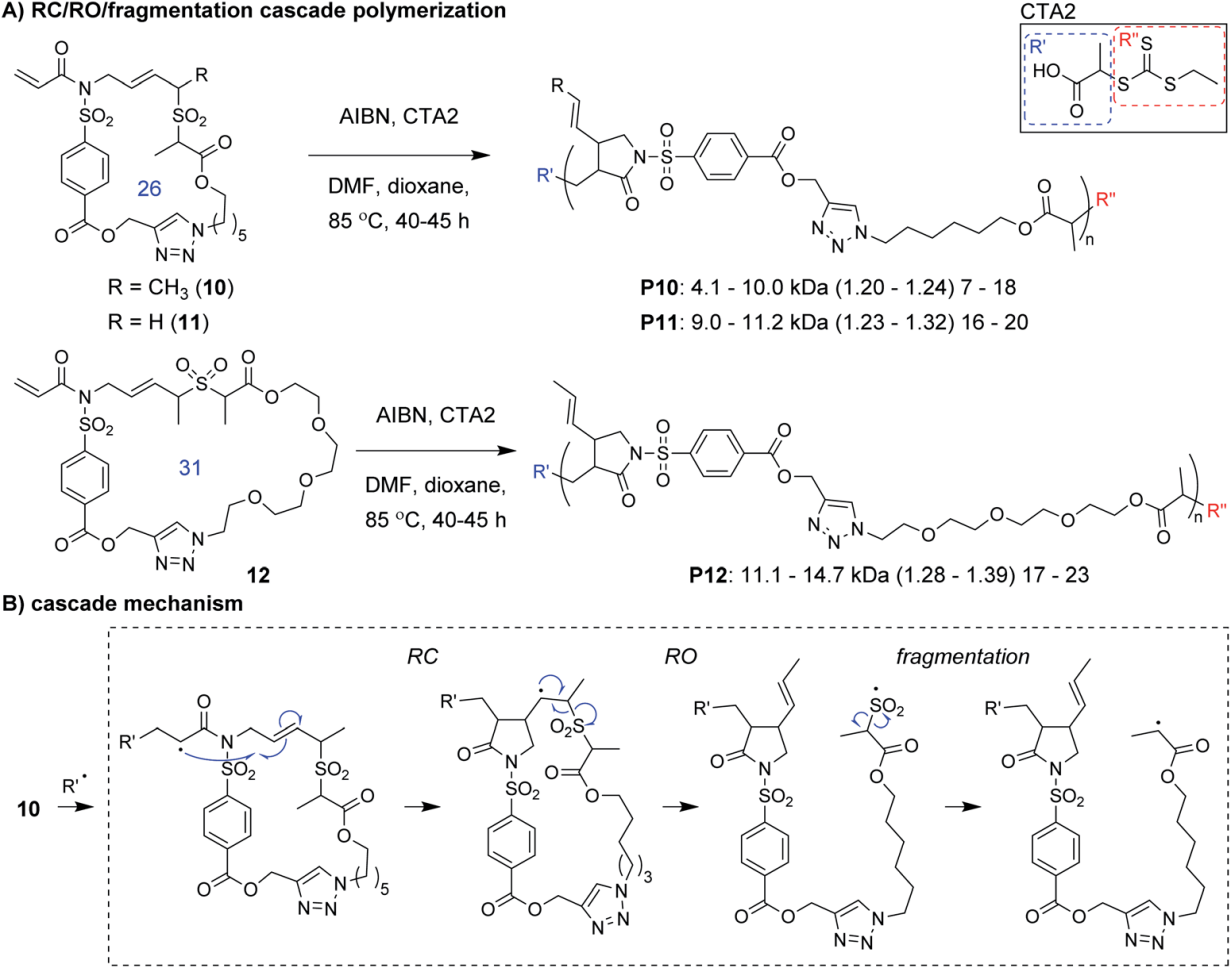
(A) Ring-opening/ring-closing/fragmentation cascade polymerization of macrocyclic monomers (ring sizes shown in blue). (B) Mechanism of the cascade sequence. The structure of CTA2 is shown in the solid box. Labels under the polymer structures = polymer label: *M*_n_ (*Đ*) DP. Abbreviations: DMF = dimethylformamide.

Niu and coworkers expanded upon this chemistry by developing new monomers in which the allylic sulfone was fused with a 1,6-diene motif (*e.g.*, **10–12**, Fig. 4A).^34^ With this modification, monomers underwent a RC/RO/fragmentation (extrusion of SO_2_) cascade to again form a carbon radical which could undergo reversible deactivation (Fig. 4B). Using RAFT polymerization conditions, a 26-membered macrocyclic monomer (**10**) gave polymers with *M*_n_ values up to 10 kDa and *Đ* values less than 1.24. Another monomer (**11**), which yields polymers with pendant vinyl groups (used as handles for post-polymerization functionalization), gave similar results. Furthermore, a 31-membered macrocyclic monomer (**12**) gave **P12** with *M*_n_ values up to 14.7 kDa and *Đ* values less than 1.39. This polymer was also shown to undergo rapid (less than 1 min) and complete degradation to small molecules under basic conditions. A significant limitation to this new class of monomers was that they underwent a side reaction at higher conversion (an acryloyl elimination with unknown mechanism, giving non-polymerizable side products), which highlights the difficulty of designing monomers that can undergo efficient cascade polymerization. While it was claimed that the resulting byproducts did not get incorporated into the polymer, they likely contributed to the slow polymerization and low molecular weight values. Nevertheless, MALDI-MS and NMR spectroscopy were used to support the repeat unit structure and conservation of the RAFT chain ends. The latter was exploited for the preparation of a block copolymer (**P10-b-P12**, *M*_n_ of 10.1 kDa and *Đ* of 1.35).

### Ionic cascade polymerizations

**Fig. 5:**
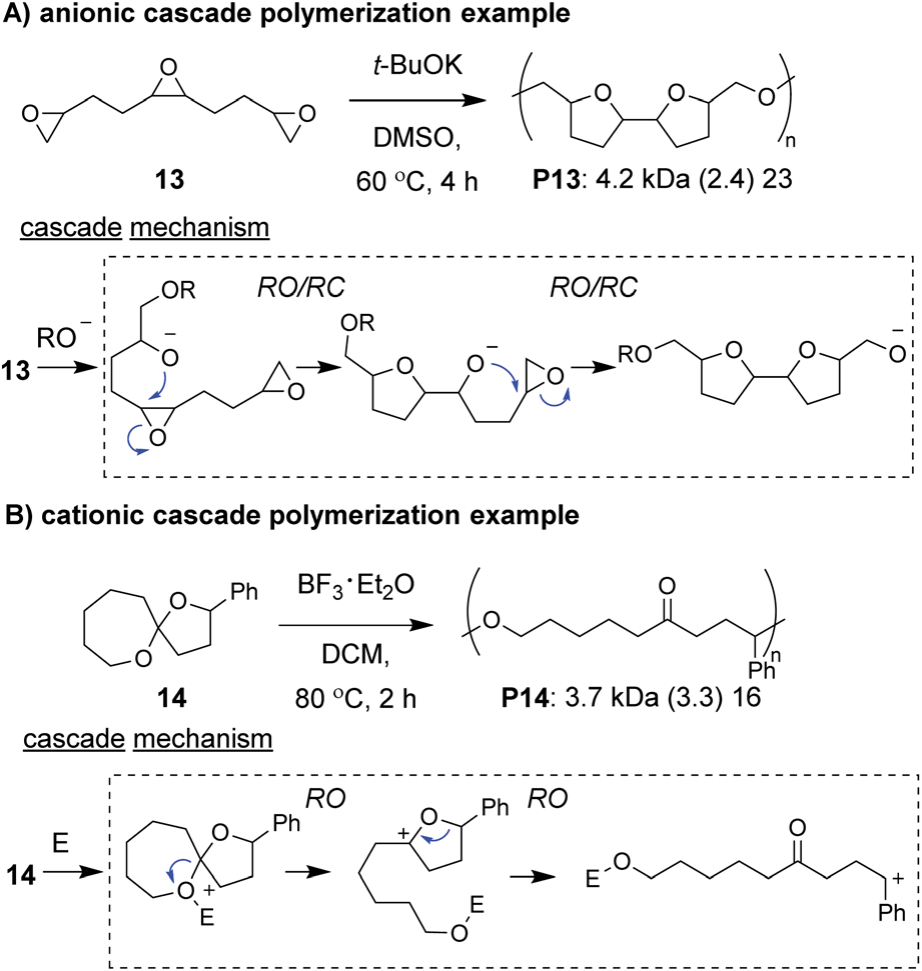
Examples of cascade polymerizations with (A) anionic and (B) cationic mechanisms. Labels under the polymer structures = polymer label: *M*_n_ (*Đ*) DP. Abbreviations: E = electrophile. DMSO = dimethylsulfoxide. DCM = dichloromethane.

Anionic and cationic cascade reactions are among the oldest and most developed subsets of small molecule cascade reactions.^1,9^ Cascade polymerizations involving anionic or cationic mechanisms, however, are much less common than those with radical mechanisms, although it is not clear why this is the case. In general, it appears that ionic cascade polymerizations do not suffer from the same selectivity issues often observed in radical cascade polymerizations. For instance, a triepoxide monomer (**13**, Fig. 5A) was shown to undergo a back-to-back simultaneous RO/RC anionic cascade, without evidence of mono- or di-epoxide pendant groups (which would result from propagation before completion of the cascade sequence).^35^ Additionally, a spiroketal monomer (**14**, Fig. 5B) was shown to undergo a cationic RO/RO cascade, without evidence of any single RO repeat units.^36^ The “double ring-opening” cascade polymerization *via* an anionic mechanism has also been demonstrated.^37,38^ Unfortunately, most ionic cascade polymerizations yield low molecular weight polymers, and only recently have higher molecular weights been obtained.

**Fig. 6:**
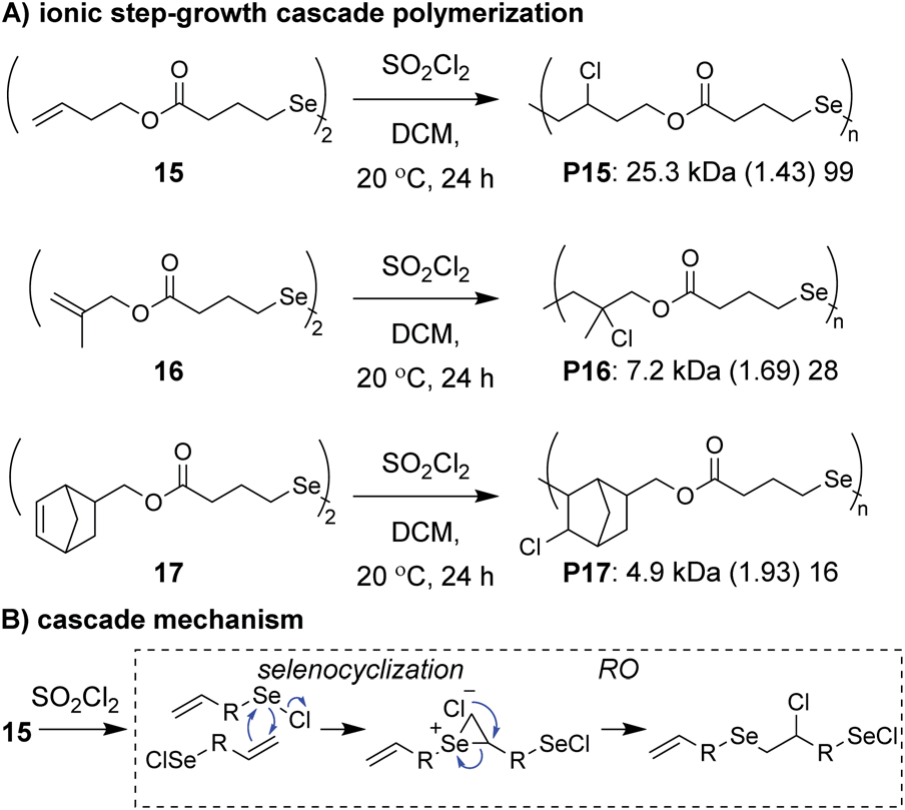
(A) Ionic step-growth cascade polymerization of diselenide monomers. (B) Mechanism of the ionic cascade sequence. Labels under the polymer structures = polymer label: *M*_n_ (*Đ*) DP.

Zhu, Pan, and coworkers developed a step-growth cascade polymerization involving cationic intermdiates.^39^ Diselenide monomers (*e.g.*, **15–17**, Fig. 6A) were treated with sulfuryl chloride to form two equivalents of a reactive selenenyl chloride intermediate, which behaved as an AB-type monomer. Specifically, the selenenyl chloride underwent rapid selenocyclization with the alkene of another monomer or polymer chain, followed by RO by the chloride ion to form the β-halo selenide linkage between repeat units (Fig. 6B).^40^ Monomers containing various terminal (*e.g.*, **15** and **16**) and internal (*e.g.*, **17**) olefins readily underwent polymerization, achieving *M*_n_ values between 3 and 34 kDa. The low molecular weight values for monomers **16** and **17** were attributed to sterically hindered olefins or poor monomer purity, respectively. An important attribute of these new polymers was that they could be degraded under relatively mild conditions. For instance, polymers were shown to undergo moderate decreases in molecular weight with 10 min of exposure to UV irradiation or HCl and essentially complete depolymerization within 10 min of exposure to NaOH or H_2_O_2_, highlighting their potential as new stimuli-responsive materials. The concept of using a halide ion for RO during propagation was also recently applied to chain-growth polymerizations (non-cascades) using the same selenyenyl chloride chemistry,^41^ or *via* the “halide rebound polymerization” developed by Gutekunst and coworkers.^42^ These polymerizations may represent potential platforms from which new ionic cascade polymerizations can be developed, including those which proceed in a controlled and living manner.

### Metathesis cascade polymerizations

**Fig. 7:**
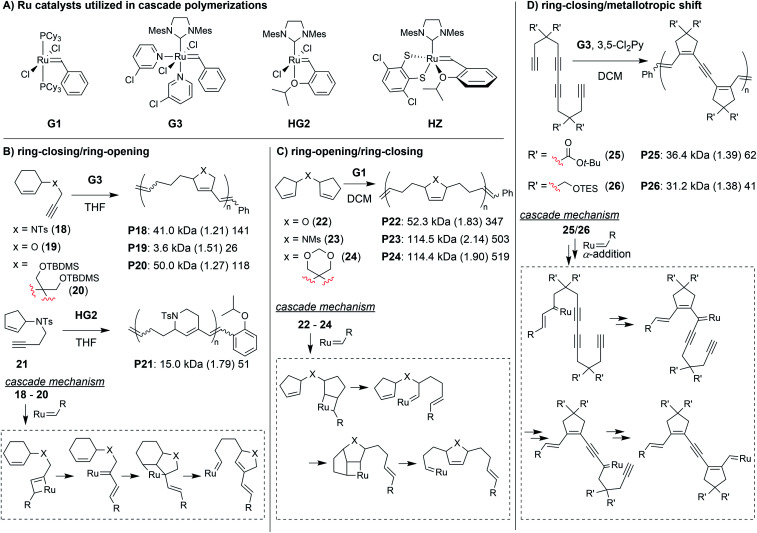
(A) Ru catalysts used in metathesis-based cascade polymerizations. The primary cascade sequences in metathesis-based cascade polymerizations are based on (B) RC/RO, (C) RO/RC, and (D) various combinations of RC and MS. Labels under the polymer structures = polymer label: *M*_n_ (*Đ*) DP. Abbreviations: Ts = tosyl. TBDMS = *t*-butyldimethylsilyl. TES = triethylsilyl. Ms = mesyl. THF = tetrahydrofuran. 3,5-Cl_2_Py = 3,5-dichloropyridine.

Transition metal-mediated cascade reactions have been extensively explored in small molecule and natural product synthesis.^43–47^ Several of these reactions have been successfully applied to cascade polymerizations. Double cyclization polymerizations,^48–50^ and various combinations of cyclopolymerization with chain walking (typically with Pd or Ni catalysts)^51–53^ have already been extensively reviewed in the literature.^54,55^ Metathesis-based cascade polymerizations (with Ru catalysts) have also been briefly reviewed.^54^ Recently, multiple new examples of metathesis-based cascade polymerizations have emerged, and they will be the focus of this section. The continued development of these polymerizations has been driven by the combination of unique monomer designs and powerful Ru catalysts (Fig. 7A). Well-defined and user-friendly Ru catalysts have enabled a wide range of powerful polymerization techniques,^56–58^ thus, it is not surprising that they have also helped enable highly efficient cascade polymerizations. The primary cascade sequences utilized in metathesis-based cascade polymerizations are: (1) RC/RO (Fig. 7B), (2) RO/RC (Fig. 7C), and (3) various combinations of RC and 1,3-metallotropic shifts (MS, Fig. 7D).

One of the first cascade polymerizations developed in our research group utilized a RC/RO cascade with monomers bearing terminal alkynes and internal cyclic alkenes (Fig. 7B).^59–61^ Monomers with a nitrogen linker between the alkyne and cyclic alkenes gave polymers with 5- or 6-membered rings in the polymer backbone (*e.g.*, **P18** and **P21**), using Grubbs third-generation (**G3**) or Hoveyda-Grubbs second-generation (**HG2**) catalysts, respectively. While bulky sulfonamide linkers (as in **18**) gave good polymerization performance, mono-substituted carbon and oxygen linkers did not polymerize under the same conditions (relatively slow and uncontrolled polymerizations could be achieved at much lower concentrations). Fortunately, by using di-substituted carbon linkers with bulky substituents (as in **20**), we could also achieve controlled polymerization *via* enhancement of the Thorpe–Ingold effect (large substituents facilitating RC) and suppression of deleterious side reactions such as chain transfer.^61^ In order to support our proposed cascade mechanism (shown in Fig. 7B), we prepared oligomeric **P18** in which the end groups could be readily observed by ^1^H NMR spectroscopy. Comparison to small molecule controls confirmed that the phenyl group (from **G3**) was being incorporated into the polymer exclusively *via* the catalyst reacting with the terminal alkyne first.^60^

**Fig. 8:**
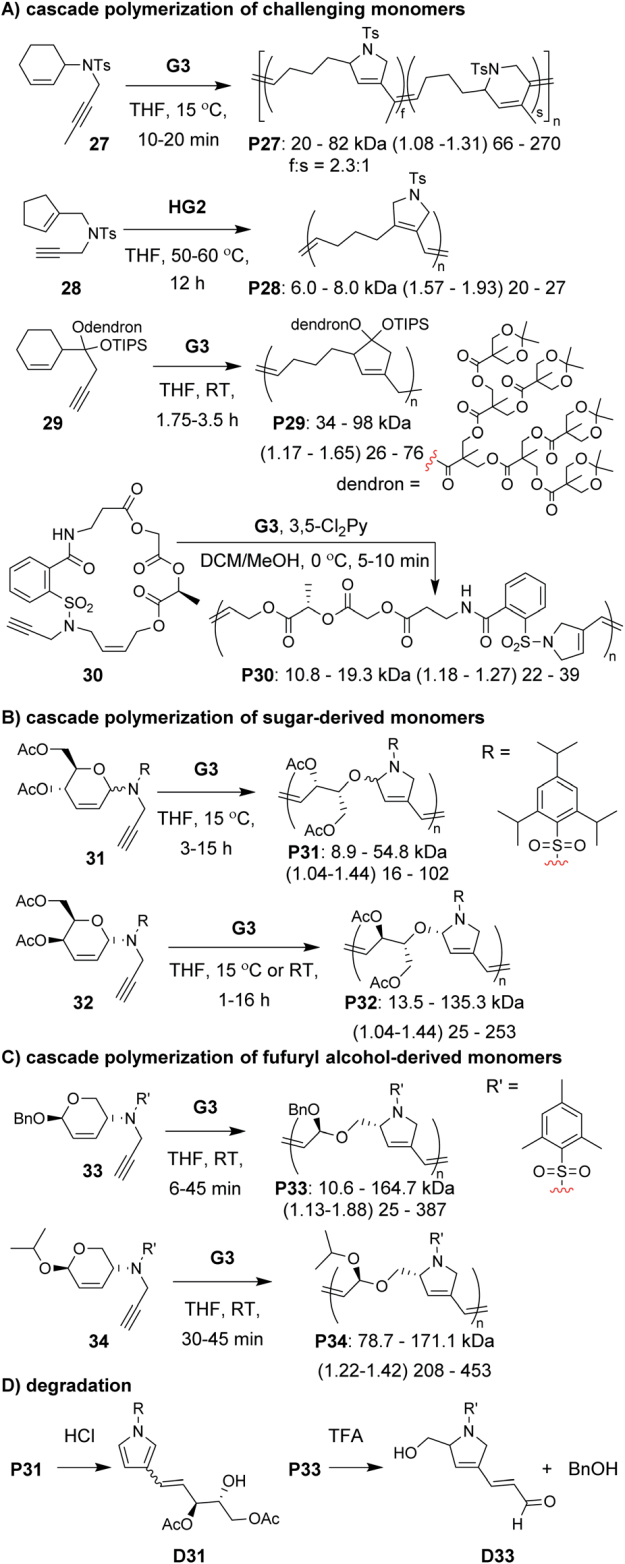
Cascade polymerizations of (A) challenging, (B) sugar-derived, and (C) furfuryl alcohol-derived monomers. (C) Degradation conditions and products. Labels under the polymer structures = polymer label: *M*_n_ (*Đ*) DP. For **P27**, the repeat unit ratio is also shown. Abbreviations: TFA = trifluoroacetic acid. RT = room temperature. TIPS = triisopropylsilyl.

A diverse group of challenging monomers could undergo the RC/RO cascade, including: monomers with internal alkynes (*e.g.*, **27**, Fig. 8A), monomers with tri-substituted alkenes (*e.g.*, **28**), and dendronized monomers (*e.g.*, **29**). One of the limitations of these examples was that the resulting polymers contained essentially all carbon backbones, which prohibited degradation. Gutekunst and Hawker addressed this issue by expanding the RC/RO cascade polymerization to challenging macrocyclic monomers containing amino acid sequences (as in **30**).^62^ They could achieve high molecular weight polymers (up to *ca.* 41 kDa) with relatively low *Đ* values (less than 1.39). Furthermore, polymers were shown to undergo complete degradation to small molecules in 2 h under methanolysis conditions. Inspired by this work, our research group sought to develop new polymers which could undergo degradation under much milder conditions.

In our efforts to expand the monomer scope of the RC/RO cascade polymerization and obtain degradable polymers, we developed new sugar-based monomers.^63^ Specifically, we synthesized monomers from d-glucose (*e.g.*, **31**, Fig. 8B) and d-galactose (*e.g.*, **32**) and showed that they underwent efficient cascade polymerization to give polymers with hemiaminal ether linkages in the backbone. As previous monomers contained unfunctionalized cycloalkene rings, we found that the stereochemistry of the tethered alkyne had little influence on monomer reactivity. This, however, was not the case when using sugar-based cycloalkenes. Presumably, the substituents on the ring provided additional steric interactions which slowed catalyst approach to the monomer or slowed the RC step. Thus, even though all monomers with bulky sulfonamides underwent controlled polymerization, the best performing monomer was **32**, in which the alkyne substituent was tethered to the opposite face of the ring relative to the other cycloalkene substituents (*i.e.*, α-stereochemistry at the anomeric carbon). For this monomer, we could obtain up to 155 kDa polymer with dispersity values between 1.04 and 1.44. The living nature of this polymerization was also supported by the preparation of block copolymers utilizing different sugar-based monomers. Gutekunst and coworkers were also working (simultaneous to our research group) to develop degradable polymers from RC/RO cascade polymerizations. Specifically, they developed monomers derived from furfuryl alcohol (*e.g.*, **33** and **34**, Fig. 8C) which gave polymers with acetal linkages in the polymer backbone.^64^ They also obtained their best results when using a bulky sulfonamide linker tethered to the opposite face of the ring relative to the other cycloalkene substituent. With their best performing monomer (**34**), they could achieve molecular weights up to 171 kDa with *Đ* values of 1.42 or less. Polymers from both of these studies (**P31–34**) where shown to undergo degradation under mild conditions. For example, both **P31** and **P33** underwent complete degradation to small molecules, **D31** and **D33**, respectively (Fig. 8D). While degradation of **P33** to **D33** was complete in *ca.* 24 h with 5 equivalents of trifluoroacetic acid (per repeat unit), degradation of **P31** to **D31** occurred in less than 10 min with *ca.* 0.6 equivalents of HCl (per repeat unit), presumably due to the more sensitive hemiaminal ether in the backbone and aromatization of the heterocycle serving as a strong driving force for degradation.

The next class of polymerization we will discuss is the RO/RC metathesis cascade polymerization, which was first studied by Chen, Luh, and coworkers.^65^ They utilized bisnorbornenes tethered by long flexible linkers and found that, under appropriate conditions, they could avoid cross-linking and achieve a linear polymer (*M*_n_ of 7.8 kDa and *Đ* of 1.2) *via* cascade polymerization. Our research group expanded upon this work by developing biscyclopentadiene monomers (*e.g.*, **22–24**, Fig. 7C) which underwent cascade polymerization.^66^ A thorough kinetics study revealed that Grubbs first-generation catalyst (**G1**) was the optimal catalyst (in spite of its generally lower reactivity compared to other Grubbs catalysts), likely due to the smaller ligands which helped promote productive metathesis pathways.^67^ However, the narrow monomer scope and low turnover numbers (TON, less than 250) motivated us to further refine the monomer structures.

**Fig. 9:**
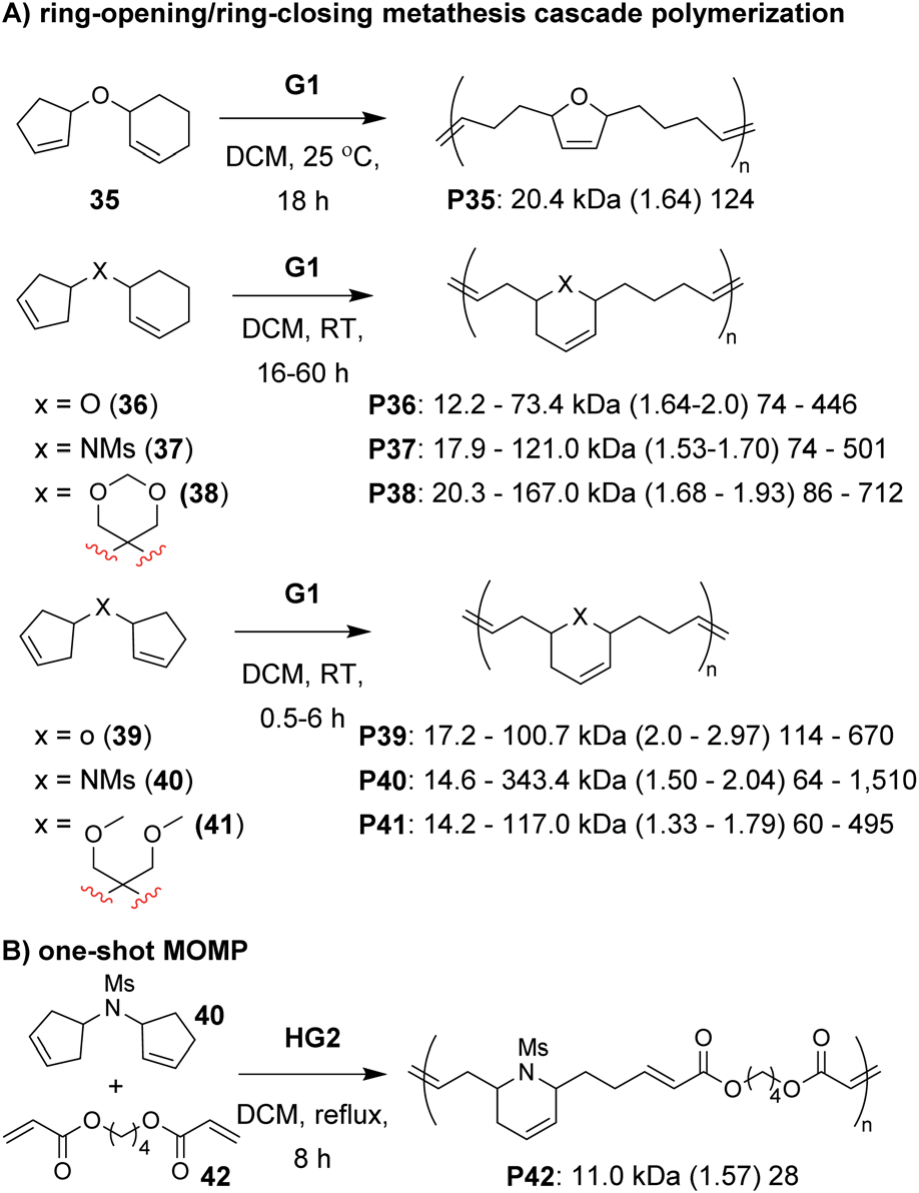
(A) Cascade polymerization of biscycloalkene monomers. (B) One-shot multiple-olefin metathesis polymerization (MOMP) combining RC, RO, and cross-metathesis reactions to achieve alternating polymers. Labels under the polymer structures = polymer label: *M*_n_ (*Đ*) DP.

Recently, we explored several new monomer designs in the RO/RC cascade polymerization of biscycloalkene monomers (*e.g.*, **35–41**, Fig. 9A).^68^ We first looked at biscyclohexene monomers, but found that they did not undergo polymerization. Exchanging one of the cyclohexenes for a cyclopentene (*e.g.*, **35**) enabled polymerization, but the conversions for monomers with carbon or nitrogen linkers were low (less than 55%). We envisioned that we could enhance the thermodynamic gain of the polymerization by forming polymers with more stable 6-membered rings in the polymer backbone. By moving the double bond within the cyclopentene ring (*e.g.*, **36–38**), we found the polymers underwent much improved polymerization with TON up to 940. Our last modification was to exchange the remaining cyclohexene with a cyclopentene with higher ring strain (*e.g.*, **39–41**). These monomers achieved TON up to 1940, which was nearly 8 times larger than the monomers in our previous study (*e.g.*, **22–24**). The new monomers were also highly effective as comonomers with diacrylates in multiple-olefin metathesis polymerization (MOMP, Fig. 9B), in which RC, RO, and cross-metathesis each occur simultaneously in a one-pot process to give well-defined polymers. Diacrylate monomers (*e.g.*, **42**) were incorporated into the polymer *via* cross-metathesis, giving alternating AB-type polymers. Specifically, monomers **35**, **36**, and **39–41** (and **22** from the previous study) gave polymers with between 92 and 97% AB alteration.

**Fig. 10:**
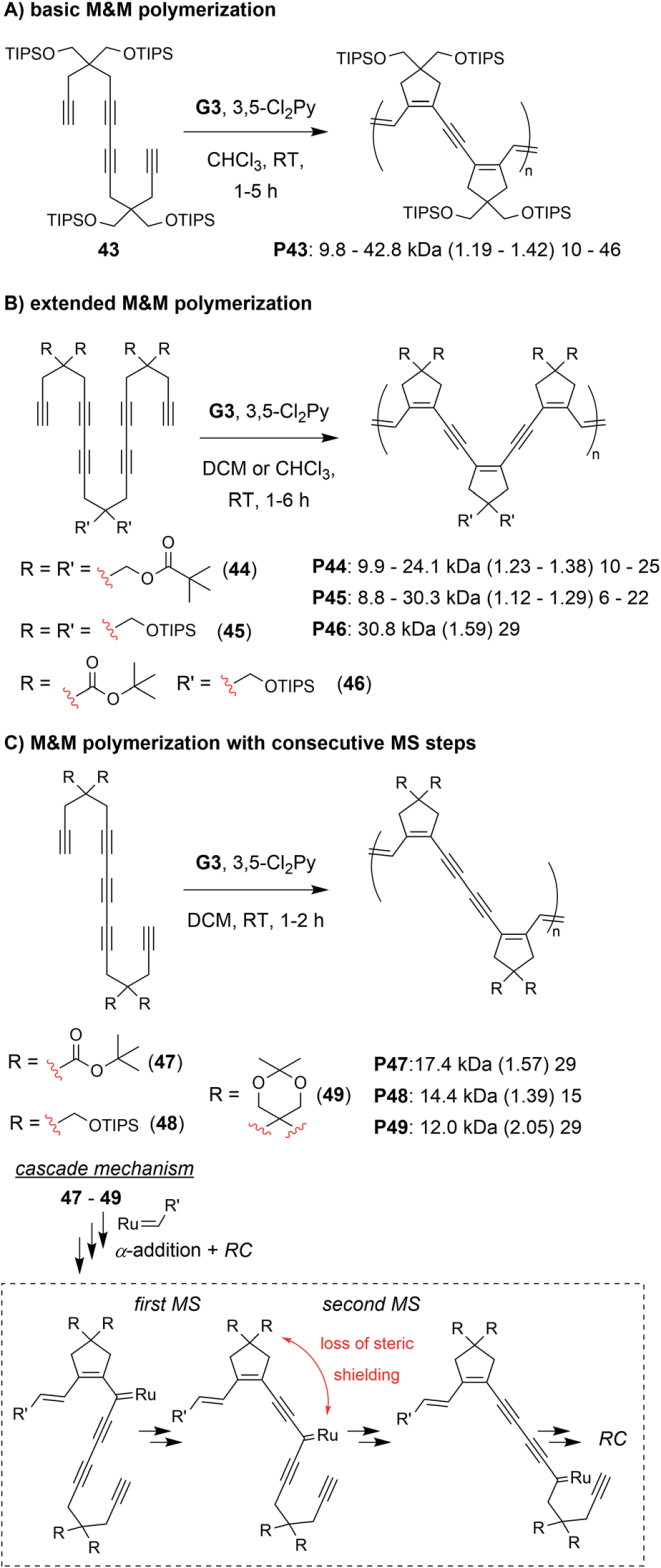
M&M polymerizations consisting of (A) RC/MS/RC, (B) RC/MS/RC/MS/RC, and (C) RC/MS/MS/RC cascade reactions. Labels under the polymer structures = polymer label: *M*_n_ (*Đ*) DP.

The final class of polymerization we will discuss is the metathesis and metallotropy (M&M) polymerization. To date, the longest cascade sequences (for metathesis-based cascade polymerizations) belong to the M&M polymerization. Specifically, this polymerization combines RC and MS to achieve fully conjugated polyenynes with 5-membered rings in the polymer backbone (Fig. 7D). Our initial studies explored the polymerization of relatively simple tetrayne monomers (*e.g.*, **25** and **26**).^69^ Controlled polymerizations were obtained by using bulky substituents (at the indicated R′ positions shown in Fig. 7D) and by adding the weakly coordinating ligand 3,5-dichloropyridine (3,5-Cl_2_Py), which in both cases slowed decomposition of the propagating carbene (these are general trends which were also observed in the cyclopolymerization of diyne monomers).^70^ We envisioned that more efficient polymerizations and even longer cascades (with greater repeat unit complexity) could be achieved by developing new monomers with bulkier substituents and longer alkyne sequences (Fig. 10).^71^ We began by exploring new bulky substituents and found that monomers with triisopropylsilyl ether substituents (*e.g.*, **43**) enabled lower dispersity values than the previous monomers, while maintaining control up to M/I of 75 (compared to M/I of 50, previously). Kinetics studies revealed that the polymerization was first-order in monomer concentration, indicating that the RC and MS steps were faster than intermolecular propagation. These results were also in good agreement with DFT calculations (conducted by our collaborators in the Baik research group).

Next, we explored the reactivity of hexaynes which underwent a total of three RC and two MS steps (*e.g.*, **44–46**, Fig. 10B; note: this monomer follows the same cascade mechanism shown in Fig. 7D, followed by an addition MS then RC step). We again found that bulky substituents were key to successful cascade polymerization. Triisopropylsilyl ether groups enabled controlled polymerization, with linear increase in *M*_n_ (up to *ca.* 30 kDa) with increasing M/I ratio, and *Đ* values less than 1.3. The living nature of the M&M polymerization was exploited by preparing a block copolymer with its first block consisting of the tetrayne monomer **43** (*M*_n_ of 9.8 kDa and *Đ* of 1.19) and second block consisting of hexayne monomer **46** (final *M*_n_ of 22.6 and *Đ* of 1.26). Finally, we explored the reactivity of pentayne monomers (*e.g.*, **47–49**, Fig. 10C) which underwent back-to-back MS steps, forming repeat units with 5-member cyclic structures separated by two alkynes. Unlike the previous monomers, increasing the steric bulk had little influence on the control of the polymerization. We rationalized that the bulky substituents were likely too far from the carbene, after the first MS step (as indicated in Fig. 10C), to provide the steric shielding effect that typically helps prevent decomposition. Regardless, the best performing monomer (**47**) gave a 17 kDa polymer with *Đ* of 1.59.

**Fig. 11:**
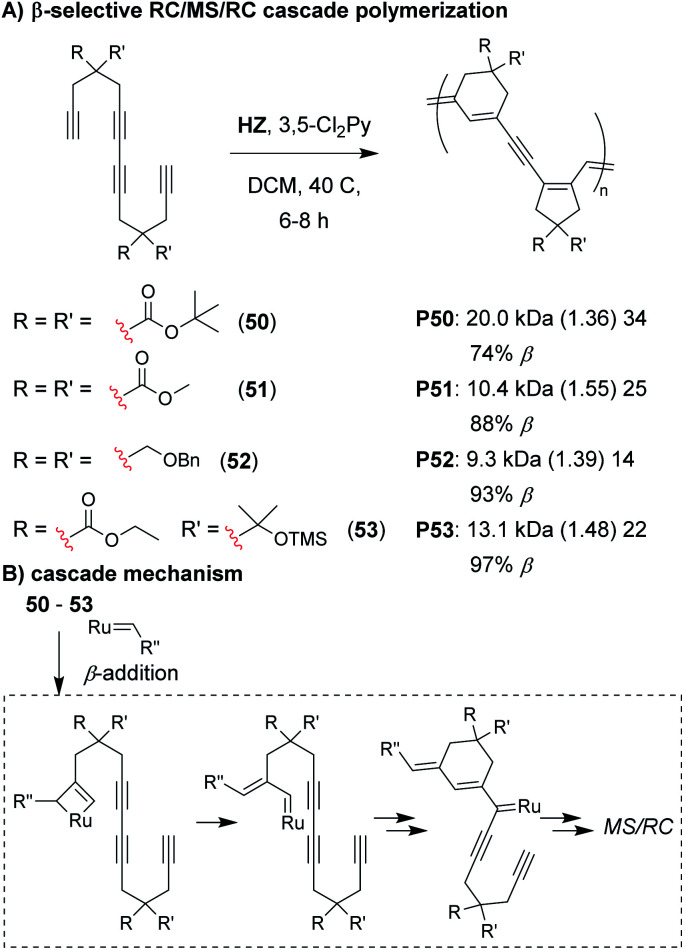
(A) β-selective M&M polymerization of tetrayne monomers. (B) Mechanism of β-addition. After the 6-membered ring formation, the cascade proceeds as shown in Fig. 7D. Labels under the polymer structures = polymer label: *M*_n_ (*Đ*) DP, the % of β repeat units is also shown. Abbreviations: TMS = trimethylsilyl.

Analogous to the cyclopolymerization of 1,6-heptadiynes, we envisioned that a Z-selective catalyst could enable propagation *via* β-addition,^72–74^ resulting in 6-membered cyclic structures after the first RC step of the M&M cascade (Fig. 11).^75^ The intermediate formed after the first RC step (Fig. 11B) would be identical to the intermediate formed after the first RC step with α-addition (as shown in Fig. 7D), thus, the rest of the cascade sequence would proceed identically and give a 5-membered ring in the second RC step. In other words, with perfect selectivity for β-addition, the resulting polymer would contain alternating 6- and 5-membered rings in the polymer backbone. We began by exploring the M&M polymerization of monomers with bulky substituents (*e.g.*, **50**) using Hoveyda's Z-selective catalyst (**HZ**).^76^ While bulky substituents enabled noticeably higher conversion at higher M/I ratios, higher β-selectivity (and better alternation) was obtained with smaller substituents (*e.g.*, **51**). NMR studies revealed that the propagating carbene species was coordinated to the internal alkyne (which was not observed in previous M&M polymerizations with **G3**), and was likely the reason for the longer polymerization times (6–8 h, compared to 1–5 h with **G3**). These results highlight how catalysts can also play an important role in the selectivity of cascade polymerizations.

### Future outlook

The development of entirely new cascade polymerizations will likely continue to be a focus of future research. As the majority of single-component cascade polymerizations are derived from RC- or RO-based polymerizations, it is unsurprising that their cascade sequences are primarily composed of RC and RO steps. This is largely a result of these reactions being able to be tuned to have high conversion, efficiency, and selectivity. For RC, this is achieved by effectively using the Thorpe–Ingold effect and forming favorable ring sizes (*e.g.*, by following Baldwin's rules). For RO, efficient reactions rely on the release of ring strain, or for non-strained rings, the formation of more thermodynamically stable functional groups. The use of RC to kinetically drive RO has also been an effective strategy for several radical- and metathesis-based polymerizations (*e.g.*, **6–12**, **18–21**, **27–34**). Fragmentation and rearrangement steps have been underutilized thus far, but have been key to the success of several polymerizations, by providing a driving force for polymerization (such as expulsion of a gas) or moving the reactive species (*i.e.*, a radical or ruthenium carbene) to another location within the monomer to react. Implementation of these and other new reactions into cascade sequences will be important to expanding the scope of novel polymers obtainable by cascade polymerizations.

As many of the cascade polymerizations presented in this review were inspired by cascade reactions in small molecules, we expect the broad collection of cascade reactions will continue to serve as inspiration for new polymerization methodologies. Multi-component cascade polymerizations should also be a source for inspiration. Through creative monomer design (*e.g.*, tethering the necessary reactive moieties), some of these polymerizations might be adapted into well-controlled chain-growth cascade polymerizations. Similarly, taking inspiration from the palladium template-mediated double radical cyclization of styrenyl monomers,^77^ template polymerizations might be utilized to non-covalently bring the reactive moieties of the monomer in close proximity to enable cascade polymerization.

Another important area of future research will be the continued expansion of known cascade polymerizations. This includes optimizing monomer structures and introducing new functional groups to increase the length of cascades and yield polymers with enhanced complexity or functionality. A good example of this was the development of the 1,6-hexadiene-allylic sulfone fusion monomers (Fig. 4) by Niu and coworkers. The application of new polymerization conditions to cascade monomers to achieve different selectivities or more controlled polymerizations is also important. Miyake and coworkers' implementation of photoredox chemistry, for instance, enabled unprecedented control over the polymerization of vinyl cyclopropanes (Fig. 2). Combining both of these approaches will likely be necessary to fully unlock the potential of a given cascade monomer class, as we have attempted to do with the M&M polymerization. By treating our multi-yne monomers as a modular platform, we could introduce new alkynes into the monomer to increase the cascade sequence length or change the sequence order (Fig. 10), and we could use different catalysts to introduce new functionalities (6-membered rings) without changing the monomer structure. Despite these advancements, many of the known cascade polymerizations still suffer from loss of polymerization performance when targeting higher M/Is, and thus, the polymers often have limited DPs (the relatively high molecular weight in some cases is due to high repeat unit molecular weight) or broad dispersity. Improving performance should continue to be an important topic to pursue.

One of the most exciting aspects of cascade polymerizations is the potential to develop unique polymers with interesting properties. With a few exceptions, however, the properties of many new polymers have not been explored in depth. For instance, thermal properties (*e.g.*, glass transition and decomposition temperatures) have often been explored, but mechanical properties have not. This is likely a limitation of the current scale of cascade polymerizations. More time should be dedicated to developing multi-gram scale polymerizations which can produce enough material to explore various processing techniques (*e.g.*, compression molding, melt extrusion, 3D printing, *etc.*) and enable characterization of mechanical properties (*e.g.*, stiffness, hardness, toughness, *etc.*). Basic optical properties have been sometimes explored (*e.g.*, optical rotation, UV-vis absorption, *etc.*), but conjugated polymers, for instance, would be well-served by implementing them in electronic devices and studying other (device-specific) optoelectronic properties. Another commonly explored property is degradability, but we are unaware of any exploration of environmental or bio-degradability (or biocompatibility for that matter) of cascade polymers. Delving deeper into these properties will be critical to identifying and optimizing these polymers for potential applications.

## Conclusions

Cascade polymerizations provide access to polymers with unique chemical structures and significantly expand the scope of how synthetic chemists can make polymers. In the last couple years, significant progress has been made for polymerizations utilizing radical- and metathesis-based mechanisms. For the former, highly selective cascade reactions have enabled polymers with well-defined structures and high molecular weight. For the later, the monomer scope and polymerization performance for known cascade reactions has been significantly expanded, and new methods to extend the length of cascade sequences have been developed. These advances have largely come about by thoughtful monomer design and implementation of controlled polymerization conditions. As progress for ionic cascade polymerizations has lagged in recent years, this still remains an important area for future study, in addition to identifying and optimizing cascade polymers for specific applications. We expect that the popularity of cascade polymerizations will continue to grow in the coming years as current methodologies are implemented and new methodologies are developed.

## Conflicts of interest

There are no conflicts to declare.
